# Delta Opioid Receptor Signaling Promotes Resilience to Stress Under the Repeated Social Defeat Paradigm in Mice

**DOI:** 10.3389/fnmol.2018.00100

**Published:** 2018-04-06

**Authors:** Mathilde S. Henry, Kanchan Bisht, Nathalie Vernoux, Louis Gendron, Angélica Torres-Berrio, Guy Drolet, Marie-Ève Tremblay

**Affiliations:** ^1^Axe Neurosciences, Centre de Recherche du CHU de Québec—Université Laval, Québec, QC, Canada; ^2^Centre de Recherche du CHU de Sherbrooke and Institut de Pharmacologie de Sherbrooke, Université de Sherbrooke, Sherbrooke, QC, Canada; ^3^Département de Pharmacologie-Physiologie, Université de Sherbrooke, Sherbrooke, QC, Canada; ^4^Quebec Pain Research Network, Sherbrooke, QC, Canada; ^5^Integrated Program in Neuroscience, McGill University, Québec, QC, Canada; ^6^Département de Psychiatrie et Neurosciences, Université Laval, Québec, QC, Canada; ^7^Département de Médecine Moléculaire, Université Laval, Québec, QC, Canada

**Keywords:** enkephalin, opioid receptor, chronic stress, resilience, oxidative stress, electron microscopy

## Abstract

The adaptation to chronic stress is highly variable across individuals. Resilience to stress is a complex process recruiting various brain regions and neurotransmitter systems. The aim of this study was to investigate the involvement of endogenous opioid enkephalin (ENK) signaling in the development of stress resilience in mice. The translational model of repeated social defeat (RSD) stress was selected to mimic the unpredictable disruptions of daily life and induce resilience or vulnerability to stress. As in humans, adult C57BL/6J mice demonstrated a great variability in their response to stress under this paradigm. A social interaction (SI) test was used to discriminate between the phenotypes of resilience or vulnerability to stress. After social defeat, the expression levels of ENK mRNA and their delta opioid receptors (DOPr) were quantified in the basolateral amygdala (BLA) and BLA-target areas by *in situ* hybridization. In this manner, ENK mRNA levels were found to decrease in the BLA and those of DOPr in the ventral hippocampus (HPC) CA1 of vulnerable mice only. Stimulating the DOPr pathway during social defeat by pharmacological treatment with the nonpeptide, selective DOPr agonist SNC80 further induced a resilient phenotype in a majority of stressed animals, with the proportion of resilient ones increasing from 33% to 58% of the total population. Ultrastructural analyses additionally revealed a reduction of oxidative stress markers in the pyramidal cells and interneurons of the ventral HPC CA1 upon SNC80 treatment, thus proposing a mechanism by which ENK-DOPr signaling may prevent the deleterious effects of chronic social stress.

## Introduction

The survival of an individual relies on its adaptation to living conditions in constant evolution. The response to chronic stress is highly variable from one individual to another. Resilience is defined as “the process of adapting well in the face of adversity, trauma, tragedy, threats or even significant sources of threat” (The American Psychological Association). Heterogeneity in the response to stress suggests that resilience is a complex neurobiological process that emerges from a multitude of gene-environment interactions. Several mechanisms have been proposed to underlie the inter-individual difference in resilience or vulnerability to stress.

Numerous studies support an important contribution of endogenous ENKs in the modulation and regulation of stress responses. The potential role of ENKs in stress resilience is suggested by their extensive distribution in the basal forebrain, especially the prefrontal cortex, nucleus accumbens, dorsal striatum, ventral tegmental area, hippocampus (HPC) and amygdala (Le Merrer et al., 2009). The opioid enkephalin (ENK) is critical to maintain hedonic and emotional balance, as it attenuates endocrine, autonomous and stress-induced behavioral responses (Bali et al., 2015; Henry et al., 2017). We have recently shown how ENK transmission is central to the neurobiology of stress resilience (Bérubé et al., 2013, 2014; Henry et al., 2017). Expression levels of ENKs mRNA were specifically decreased in the basolateral amygdala (BLA) of vulnerable rats, while ENK knockdown in the BLA reproduced a vulnerability phenotype in rats (Bérubé et al., 2013, 2014). *In vivo*, ENK have a preferential affinity for two types of opioid receptors: mu opioid receptor (MOPr) and delta opioid receptor (DOPr; Mansour et al., 1986). Genetic inactivation of ENK as well as DOPr in mice induces a high level of anxiety whereas mice deficient for MOPr display reduced anxiety (Filliol et al., 2000; Bilkei-Gorzo et al., 2004). Moreover, DOPr antagonists promote anxiety while treatments with agonists exert the opposite effect in rats (Perrine et al., 2006).

Recently, chronic psychological stress during childhood, post-traumatic stress disorder (PTSD), and several other stress-related conditions, were found to be associated with increased levels of oxidative stress (Miller et al., 2011; Miller and Sadeh, 2014). Conversely, reducing anxiety-like behavior in mice using benzodiazepine treatment prevents the exacerbated production of reactive oxygen species that is induced by chronic restraint stress (Núñez et al., 2011). Oxidative stress corresponds to the disturbed balance between free radicals and antioxidant molecules. Higher levels of toxic reactive species impair cellular functions by affecting lipids, proteins, as well as nucleic acids (Czerska et al., 2015). Moreover, the neuroprotective effects of DOPr have been linked to reduced oxidative stress (Ma et al., 2005; Wallace et al., 2006; Yang et al., 2009). In particular, Wallace et al. (2006) demonstrated that DOPr activation, using [D-Pen^2^, D-Pen^5^)]-Enkephalin (DPDPE) or SNC80, counteracts the oxidative stress induced by human immunodeficiency virus toxins *in vitro*. In addition, DOPr pharmacological activation using intraperitoneal injection of [D-Ala^2^, D-Leu^5^]-Enkephalin (DADLE) is protective against brain hypoxic injury in rats, resulting in an attenuation of oxidative damage exerted through increased activity of antioxidant enzymes (Yang et al., 2009). On the opposite, morphine (MOPr agonist) administration in rat spinal cord exacerbates oxidative stress (Ozmen et al., 2007).

The purpose of this study was to investigate the functional contribution of ENK/DOPr pathway to the emergence of stress resilience in mice, using a combination of stress paradigm, behavioral testing, molecular biology and ultrastructural analyses. Social stress was specifically targeted considering that social interactions (SIs) represent the main source of stress from the environment. We performed the repeated social defeat (RSD) paradigm, which results in either vulnerability or resilience to stress. This variance in behavioral outcome makes it an excellent model to study the underlying mechanisms of stress resilience (Krishnan et al., 2007; Golden et al., 2011; Duclot and Kabbaj, 2013). Our results first confirmed the proportion generally obtained for resilient and vulnerable phenotypes in mice, as mentioned in Golden et al. (2011). Indeed, 33% of mice displayed a resilient phenotype while the rest of them showed vulnerability to stress. The expression levels of ENK and DOPr mRNA in the BLA and the HPC, respectively, were measured in animals exposed to RSD and their non-stressed controls. The mRNA levels of ENK were found to be decreased in the BLA and those of DOPr in the ventral HPC CA1 of vulnerable mice compared to control and resilient ones. To assess the functional impact of this pathway on stress resilience under RSD, we next hypothesized that activation of DOPr signaling by SNC80 treatment throughout RSD could promote resilience. SNC80 is a nonpeptide, selective DOPr agonist able to cross the blood brain barrier (Bilsky et al., 1995). This compound was previously shown to induce DOPr-mediated anxiolytic (Saitoh et al., 2004, 2005), antidepressive (Broom et al., 2002), as well as analgesic effects in rodents (Bilsky et al., 1995). In our experiments, administration of SNC80 induced a resilient phenotype in a majority of stressed animals, with the proportion of resilient ones increasing from 33% to 58% of the total population. We lastly tested the hypothesis that DOPr signaling enhances resilience to chronic social stress by preventing oxidative damage to excitatory and inhibitory neurons of the ventral HPC CA1. Ultrastructural analyses were thus conducted using transmission electron microscopy (TEM), which revealed a major effect of SNC80 on dampening oxidative stress, both in interneurons and pyramidal cells, when quantifying changes in several of their features. Indeed, SNC80 reduced the proportion of cells with cytoplasmic/nucleoplasmic condensation, nuclear indentation, as well as endoplasmic reticulum (ER) and Golgi apparatus dilation, among other markers of oxidative stress.

## Materials and Methods

### Animals

All experiments were performed under approval of the institutional animal ethics committees, in conformity with the Canada Council on Animal Care guidelines (animal protocols n° 2014-037, 2016-073 and 242-14B). Male mice were used as detailed below. C57BL/6J mice were acquired from Jackson Laboratories (Bar Harbor, ME, USA) and all other mice from Charles River (St. Constant, QC, Canada). The animals were housed under a 12 h light-dark cycle at 22–25°C with free access to food and water.

### Repeated Social Defeat

First, CD1 retired breeders (4–6 months old) were screened for their level of aggressiveness in presence of naïve C57BL/6 mice (8–20 weeks old) for 3 days. C57BL/6J mice (7–8 weeks old) were randomly assigned to social defeat (*N* = 30, for 3 cohorts; see Supplementary Figure S1 for schematic representation) or control groups (*N* = 26, 3 cohorts). Mice from the social defeat group (intruders) were subjected to 10 consecutive days of stress as described in Golden et al. (2011). In brief, intruders were daily housed with an aggressive CD1 mouse in its home cage for 5 min of interaction. For the next 24 h, i.e., until the next defeat, the intruders were housed on the other side of the cage, separated by a perforated divider allowing for visual, olfactory and auditory contact. Each intruder was exposed to the same group of resident CD1 mice, in a different order, and to a different resident daily. The experimental mice were weighed every 2 days and their health status monitored carefully. Control C57BL/6J mice were paired-housed in defeat boxes and changed partners every day.

One day after the final defeat, both defeated and control mice underwent a SI test. At first, each mouse was placed alone for 150 s in the middle of an open-field arena (42 cm × 42 cm × 42 cm). A CD1 mouse screened for aggressiveness not previously used for defeat was then introduced in the social interaction zone (IZ) for 150 s, within a wire-mesh enclosure. Videos were recorded and analyzed with ANY-maze (Stoelting Co, Wood Dale, IL, USA). The test was performed by an observer blind to the experimental conditions. Despite the stress experience, mice still interacting with the CD1, i.e., spending more time in the IZ, were considered resilient. Vulnerable mice had a tendency to freeze in front of the CD1 and head toward the corner zones (CZs), showing social avoidance. Thus, a SI ratio was calculated as the time spent in the SI zone in the presence of a CD1 mouse divided by the time spent in the SI zone in the absence of a CD1 mouse. To discriminate between resilient and vulnerable populations, as generally done in mice, a theoretical cut-off criterion was set to 1 for the SI ratio (Berton et al., 2006; Krishnan et al., 2007; Golden et al., 2011; Menard et al., 2017). Resilient individuals presented a SI ratio superior or equal to 1, and vulnerable ones a SI ratio inferior to 1. Non-stressed controls included in the experiment had a ratio above 1 and those presenting a SI ratio below 1 were excluded, reducing the number of controls from 26 to 18 mice.

### Pharmacological Treatment

SNC80 is a selective and nonpeptidic DOPr agonist (Bilsky et al., 1995). Effectiveness of the treatment was confirmed using the forced-swim test (FST) and tail suspension test (TST) on a cohort of 32 C57BL/6 mice (7–8 weeks old) not exposed to RSD (see Supplementary Figure S1). The mice were treated with SNC80 s.c. (10 mg/kg) or administered a saline solution s.c. (0.9%) 1 h (FST) or 30 min (TST) before the test. For the FST, 6 mice per group were each placed in a transparent plastic cylinder (30 cm tall × 20 cm wide), filled with water (25°C ± 1°C, 15 cm deep), for two swimming sessions: an initial 10 min of training, followed, 24 h later, by a 6 min test session. Results were normalized to the last 4 min of test session. Videos were recorded with ANY-maze and the time spent immobile was measured by an observer blind to the experimental conditions. For the TST, 10 mice per group were suspended by the tail with adhesive tape for 6 min in a suspension chamber. An automated tail-suspension apparatus (TS100 Tail Suspension, Hamilton Kinder, CA, USA) was used to measure total time of immobility. Four animals were tested simultaneously on separate units. The last 5 min of testing were recorded. The data were clustered in 2 s periods and analyzed (Vibration Monitor software, Hamilton Kinder). Settings used in the TST experiments were as follow: threshold 0.40 Newton, off delay 40 ms, full scale 4.00 Newton.

For the RSD experiments with SNC80 treatment (N(control) = 16 and N(defeated) = 26 mice; see Supplementary Figure S1), the compound was administered for 2 days of habituation before RSD and then, 1 h prior to the defeat, on each day of social stress. SNC80 can induce epileptic seizures. Although we did not extensively study this effect, seizures were observed in approximately 80% of the mice treated with SNC80. At a dose of 10 mg/kg s.c., the mice experienced mild, epileptic-like seizures 6–8 min after the injection. The clonic seizures were observed over a period of 2–5 min after their first appearance, in accordance with what has been described by others (Chung et al., 2015). Most importantly, no signs of convulsions were observed when the animals were submitted to any of our behavioral tests (TST, FST, RSD).

### Perfusion and Tissue Preparation

The experimental C57BL/6J mice were sacrificed 1 day after the SI test. For *in situ* hybridization, 11 non-stressed control, 7 resilient and 13 vulnerable mice (see Supplementary Figure S1) were anesthetized with ketamine/xylazine (80 and 10 mg/kg, i.p.) and perfused with 0.9% saline (142.5 mM, pH 7). Brains were fixed by immersion in 4% paraformaldehyde (PFA; in 0.1 M borax buffer, pH 9.5) for 5 days at 4°C, followed by 20% sucrose in PFA/borax solution for one additional day at 4°C. Twenty micrometer-coronal slices were cut with a microtome (Leica SM 2000R) and stored at −20°C in cryoprotectant. For TEM, 3 mice per group (control, resilient, or vulnerable, treated or non-treated with SNC80; see Supplementary Figure S1) were anesthetized with sodium pentobarbital (80 mg/kg, i.p.) and perfused with 3.5% acrolein followed by 4% PFA (both in phosphate buffer—PB 100 mM, pH 7.4). Fifty micrometer-coronal sections were cut in sodium phosphate buffer (PBS; 50 mM, pH 7.4) with a vibratome (Leica VT1000S) and stored at −20°C in cryoprotectant.

### Corticosterone ELISA in Plasma

Blood samples were collected from all the experimental C57BL/6J mice through the mandibular vein, without anesthesia, 1 h prior to sacrifice. Blood was collected in a microvette tube (CB300; Sarstedt, Montréal, QC, Canada). Corticosterone levels were determined by immunoassay using a commercial ELISA kit (Cayman Chemical, Ann Arbor, MI, USA). Plates were read at 405 nm with a Microplate Reader (iMark™, Biorad, Hercules, CA, USA). Samples were diluted at 1:100 and corticosterone standards prepared. Samples concentrations were determined using a standard curve (logarithmic scale) followed by a four-parameter logistic fit analysis.

### Radioactive *in Situ* Hybridization

Slices from Bregma 1.94 mm to −3.64 mm (*The Mouse Brain in Stereotaxic Coordinates*, Paxinos and Franklin, 3rd edition) were used for *in situ* hybridization. Protocols for riboprobe synthesis and *in situ* hybridization are described in Bérubé et al. (2013). The cRNA probe directed against ENK (938bp) was generated from a plasmid targeting nucleotides −104 to +832, and the probe against DOPr (1366bp) targeted nucleotides +48 to +1412. Plasmids were generously provided by Drs. Steven L. Sabol (National Institutes of Health, Bethesda, MD, USA; Yoshikawa et al., 1984) and Mary E. Abood (Lewis Katz School of Medicine at Temple University, Philadelphia, PA, USA). Hybridization of brain slices was revealed after 18 h (ENK) or 45 h (DOPr) on KODAK BioMax MR Films (Kodak, Rochester, NY, USA) exposed with a Precision illuminator B95 (Imaging Research, St. Catharines, ON, Canada). Autoradiographic images were digitally acquired with a Retiga-2000R camera (QImaging, Surrey, BC, Canada). Optical density was quantified using ImageJ and normalized with C14 standard slides (American Radiolabeled Chemicals, St. Louis, MO, USA). Mean gray value was measured in each C14 standard corresponding to a value of radioactivity in μCi/g of tissue, and then a standard curve was calculated. The same measure of gray value was performed for each brain region and reported on the standard curve. To identify the different hippocampal regions in which DOPr mRNA levels were quantified, *The Mouse Brain in Stereotaxic Coordinates* (Paxinos and Franklin, 2nd edition) was used as a reference. Pyramidal cell layers, in which DOPr mRNAs are strongly expressed, served as boundaries to delineate the other layers.

### Electron Microscopy

#### Tissue Processing and Imaging

Brain sections from Bregma 2.92 mm to −3.52 mm were rinsed in PBS (50 mM, pH 7.4), postfixed flat in 1% osmium tetroxide, dehydrated in ascending concentrations of ethanol, treated with propylene oxide, and impregnated in Durcupan (Sigma-Aldrich, Oakville, ON, Canada) overnight at room temperature as described (Bisht et al., 2016a). After resin polymerization at 55°C for 72 h, the areas of interest containing ventral HPC CA1 were cut at 70 nm with an ultramicrotome (Leica Ultracut UC7). Ultrathin sections were collected on square-mesh grids and examined at 80kV using a FEI Tecnai Spirit G2 microscope. For analysis, 16 pyramidal cells and 10 interneurons on average, in each of *strata oriens* and *radiatum*, were randomly photographed at magnifications between 890× and 4800× using an ORCA-HR camera (10 MP; Hamamatsu). *Strata radiatum* and *oriens* were identified based on their cellular and subcellular contents, and position relative to the CA1 pyramidal layer.

#### Ultrastructural Analysis

Only neurons showing a nuclear profile above 3 μm in diameter (measured with ImageJ) were included in the analysis. Well-characterized ultrastructural features were used to assess cellular stress. Cytoplasmic and nucleoplasmic condensation (Peters et al., 1998) induces a “dark” appearance of neuronal soma, nucleus as well as dendrites and axons. Soma whose mean gray value was below 125 arbitrary units were considered as “dark”. Rough ER and Golgi apparatus dilation was identified by the expansion of lumen widths above 100 nm. Soma presenting at least one of these features were scored (Welch and Suhan, 1985; Schönthal, 2012). Nuclear membrane indentation above 600 nm (Davies et al., 1997) and lipofuscin granules recognized by their darker, granular, heterogeneous matrix and considered a hallmark of aging (Sohal and Wolfe, 1986) were also quantified. The proportion of neurons displaying at least one of each of these features was determined for each subpopulation analyzed (pyramidal cells, interneurons from *stratum oriens*, and from *stratum radiatum*).

### Statistical Analyses

Statistical significance and normality were calculated using GraphPad version 6.0 (La Jolla, CA, USA). All variables were normally distributed based on Bartlett’s test. Statistical outliers which were identified using Grubb’s test were removed from the analyses. Student *t*-test was used to assess effectiveness of SNC80 treatment. Multiple comparisons were performed by one-way ANOVA followed by Bonferroni *post hoc* analyses for behavioral characterization and *in situ* hybridization. Two-way ANOVA with Bonferroni *post hoc* analyses were used to assess the behavioral characterization as well as the effects of SNC80 treatment in other experiments. For the behavioral characterization, the two examined factors are the stress phenotype (non-stressed control, resilient or vulnerable mice) and the presence or absence of a CD1 mouse in the wire mesh enclosure during the SI test. Regarding electron microscopy (EM) analyses, the two examined factors are the stress phenotype and the presence or absence of SNC80 treatment. All data are presented as means ± standard error of the mean (SEM).

## Results

### Vulnerable Mice Display a Strong Social Avoidance Behavior Upon Repeated Social Defeat

**Figure 1:**
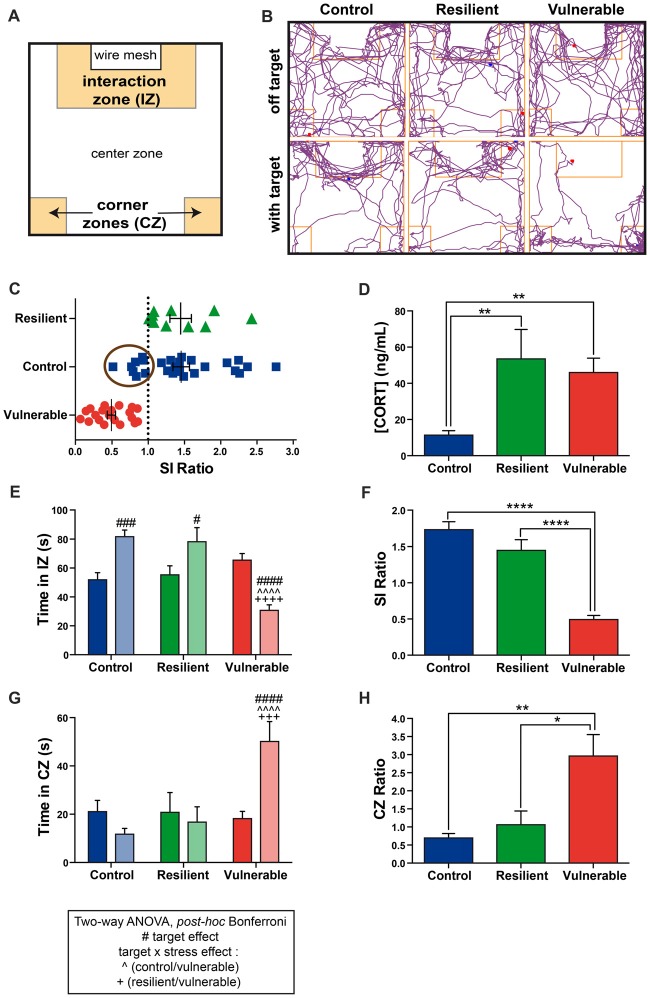
Vulnerable mice display a strong social avoidance behavior upon repeated social defeat (RSD).** (A)** Schematic overview of the social interaction (SI) arena. **(B)** Examples of movement track plots for control, resilient and vulnerable mice inside the SI test arena in the absence (off target) vs. presence of an aggressive CD1 (with target). **(C)** Distribution of SI ratio among the mouse population. N(control, C) = 26, N(resilient, R) = 10, N(vulnerable, V) = 20. Error bars are mean ± standard error of the mean (SEM).** (D)** The plasmatic corticosterone levels are increased in resilient and vulnerable mice compared to controls as measured by ELISA, confirming effectiveness of the stress paradigm. Error bars are mean ± SEM. **(E)** Control and resilient mice spend more time, and vulnerable mice less time, in the interaction zone (IZ) in the presence vs. absence of an aggressive CD1 mouse. **(F)** Vulnerable mice present a reduced SI ratio compared to control and resilient mice. **(G)** Control and resilient mice spend the same time, while vulnerable mice spend more time, in the corner zones (CZs) in the presence or absence of an aggressive CD1 mouse. **(H)** Vulnerable mice present an increased CZ ratio compared to control and resilient mice. For **(E,G)**: Two-way ANOVA, Bonferroni *post hoc* analysis, n(C) = 18, n(R) = 10, n(V) = 20,^#^*p* < 0.05, ^###^,^+++^*p* < 0.001, ^####^, ^∧∧∧∧^,^++++^*p* < 0.0001. The two analyzed factors are the stress phenotype (blue, green and red bars for control, resilient and vulnerable mice, respectively) and the presence (pale shades) or absence (dark shades) of a CD1 mouse. ^#^for target effect, for stress × target effect, ^∧^(Control vs. Vulnerable), ^+^(Resilient vs. Vulnerable). For **(D,F,H)**: One-way ANOVA, Bonferroni *post hoc* analysis, N(C) = 18, N(R) = 10, N(V) = 20, *p < 0.05, **p < 0.01, ****p < 0.0001. Error bars are mean ± SEM.

A chronic social defeat stress in mice was performed for 10 days followed by a SI test on the next day. Behavioral results are presented in Figure 1. Figure 1A presents a schematic overview of the open-field arena used for the SI test, which is divided into three main zones (interaction, corners, center). Figure 1B provides examples of movement track plots inside the SI test arena in the absence (off target) vs. presence of an aggressive CD1 in the wire-mesh container (with target). The tested mice freely explored the entire arena in the absence of a CD1 mouse (top panel), and this pattern was modified when a CD1 mouse was introduced: control and resilient mice tend to spend more time interacting with the CD1 (bottom left and middle panels) while a progression toward the CZs was generally observed with the vulnerable mice (bottom right panel). This exploration pattern was quantified, thus allowing to determine the SI and CZ ratios. Resilient mice with a SI ratio above or equal to 1 accounted for 33% of the population (10 resilient mice out of 30), whereas vulnerable mice with a SI ratio inferior to 1 accounted for 67% of the population (20 vulnerable mice out of 30; Figure 1C), as expected for the RSD paradigm in mice (Golden et al., 2011). Non-stressed control mice presenting a SI ratio below 1 (suggesting a stressed phenotype) were removed from the experiment, as shown by the encircled symbols in Figure 1C.

Two-way ANOVA was performed to assess differences in the time spent in the IZ and CZs between stress phenotypes, in the presence vs. in the absence of a CD1 mouse (Figures 1E,G). ANOVA analysis of the time spent in the IZ revealed main effects of stress phenotype (Figure 1E: *F*_(2,90)_ = 9.318, *p* = 0.0002), and interaction target by stress phenotype (*F*_(2,90)_ = 25.81, *p* < 0.0001). *Post hoc* analysis especially revealed that control and resilient mice spent more time interacting with the CD1 mouse than vulnerable ones (Figure 1E). Consequently, vulnerable individuals presented a reduced SI ratio compared to control and resilient mice (Figure 1F: *F*_(2,45)_ = 51.21, *p* < 0.0001). The time spent in the CZs showed main effects of stress phenotype (Figure 1H: *F*_(2,90)_ = 6.397, *p* = 0.0025) and interaction (*F*_(2,90)_ = 8.697, *p* = 0.0004). In presence of a CD1 mouse, vulnerable mice spent more time in the CZs than control and resilient mice without significant difference between control and resilient mice (Figure 1G). Thus, vulnerable mice had an increased CZ ratio as compared with control and resilient mice (Figure 1H: *F*_(2,44)_ = 7.738, *p* = 0.0013), indicating a strong social avoidance behavior in the SI test. These results are in accordance with the *Nature Protocol* described by Golden et al. (2011). Confirming efficacy of our stress paradigm, we found that stressed mice, whether resilient or vulnerable, presented higher plasmatic corticosterone levels than non-stressed controls (Figure 1D: *F*_(2,45)_ = 7.244, *p* = 0.0019).

### Decreased Expression Levels of ENK and DOPr mRNA Are Associated With Vulnerability to Stress

**Figure 2:**
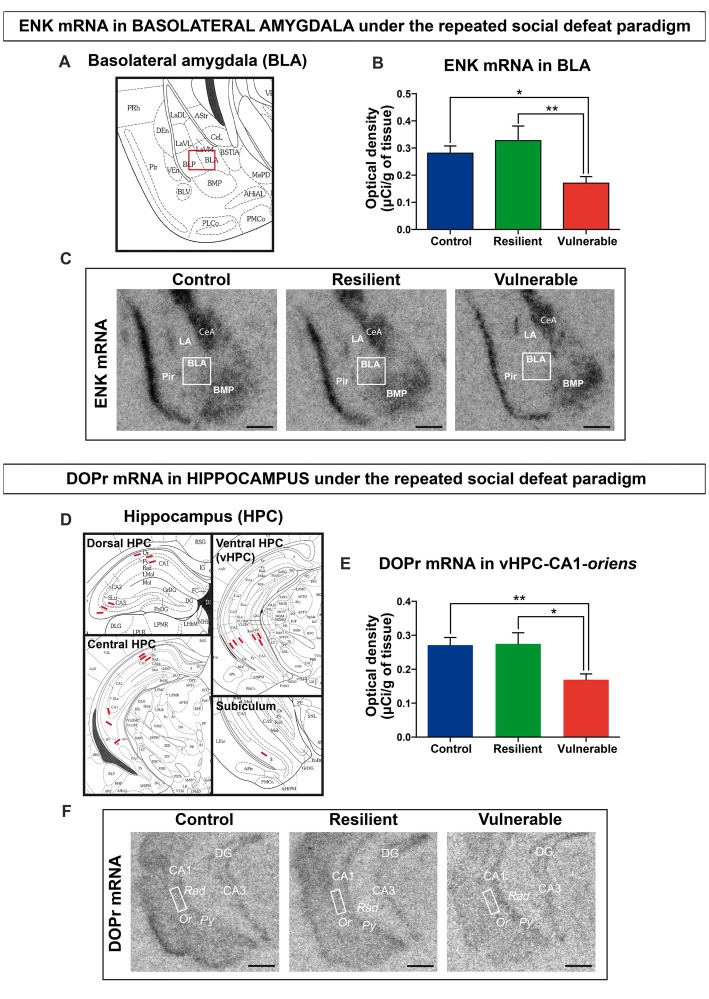
Decreased expression levels of enkephalin (ENK) and delta opioid receptors (DOPr) mRNA are associated with vulnerability to social stress.** (A)** Schematic overview of amygdala region (Bregma −1.94 mm), Image credit: Franklin and Paxinos, 2nd edition. **(B)** ENK mRNA are decreased in the basolateral nucleus of amygdala (BLA) of vulnerable mice after RSD. **(C)** Magnified view of representative autoradiographs showing the amygdalar region selected (Bregma −1.94 mm) for ENK mRNA quantification by radioactive *in situ* hybridization. Scale bar = 0.5 mm. **(D)** Schematic overview of Hippocampus (HPC) for quantification in dorsal (Bregma −1.82 mm), central (Bregma −2.54 mm) and ventral HPC (Bregma −3.08 mm), and subiculum region (Bregma −3.52 mm), Image credit: Franklin and Paxinos, 2nd edition. **(E)** DOPr mRNA are decreased in the *oriens* layer (Or) of CA1 HPC in vulnerable mice after RSD. **(F)** Magnified view of representative autoradiographs showing the ventral HPC region selected (Bregma −3.08 mm) for DOPr mRNA quantification by radioactive *in situ* hybridization. Scale bar = 0.5 mm. For **(B,E)** One-way ANOVA, Bonferroni *post hoc* analysis, N(C) = 11, N(R) = 7, N(V) = 13, *p < 0.05, **p < 0.01. Error bars are mean ± SEM. The nomenclature used is from the Mouse brain atlas, Paxinos and Franklin, 2nd edition. BMP, posterior basomedial nucleus of amygdala; CeA, central amygdala; LA, lateral amygdala; BLA, basolateral amygdala; Pir, piriform cortex; CA1/CA3, regions in HPC; DG, dentate gyrus; *Py, stratum pyramidale; Rad, stratum radiatum; Or, stratum oriens*.

To study the involvement of ENK signaling in stress resilience under the RSD, we performed radioactive *in situ* hybridization and quantified the expression levels of ENKs mRNA in BLA of control, resilient and vulnerable mice. Representative schematic of BLA is provided in Figure 2A (Bregma −1.94 mm). Representative autoradiography images from control, resilient and vulnerable mice are presented in Figure 2C. Resilience to chronic stress was previously associated with reduced ENKs mRNA levels in BLA of vulnerable rats upon social defeat, and inactivation of ENKs in the same area reproduced a vulnerability phenotype (Bérubé et al., 2013, 2014). Similarly, our current analysis revealed a significant reduction of ENKs mRNA in the BLA of vulnerable mice compared to control and resilient ones (Figure 2B: *F*_(2,28)_ = 6.491, *p* = 0.0048), without significant difference between resilient and control animals (Figure 2B). The combined results suggest conservation across rodent species of ENK involvement in the resilience to chronic social stress.

To dissect the ENK circuitry mediating stress resilience, radioactive *in situ* hybridization against DOPr was next performed. Neurons from the BLA are known to innervate the dorsolateral caudoputamen, prefrontal cortex, HPC, and several hypothalamic nuclei in rodents (Petrovich et al., 2001; Hoover and Vertes, 2007). Our analyses focused on the HPC, considering its critical regulation of the hypothalamic-pituitary-adrenal axis during stress (McEwen et al., 2015), the known interplay between the amygdala and the HPC in chronic stress response (Vyas et al., 2002), and the abundant expression of DOPr in the HPC (Erbs et al., 2012). The analyses were performed across the dorsal, central, and ventral HPC (Bregma at −2.06 mm, −2.70 mm and −3.08 mm), comparing the CA1, CA3, and ventral subiculum regions (Figure 2D). Quantitative analysis revealed that DOPr mRNA expression levels are significantly reduced in vulnerable mice vs. control and resilient animals, in only one examined region: stratum *oriens* of the CA1 from ventral HPC (Figure 2E: *F*_(2,29)_ = 7.588, *p* = 0.0022). Representative autoradiography images are shown in Figure 2F. To support specificity of the probe against DOPr, pictures for negative control are shown in Supplementary Figure S2. A table is provided in the Supplemental information presenting data from all the hippocampal regions analyzed by *in situ* hybridization (Supplementary Table S1).

### Administration of a DOPr Agonist, SNC80, Promotes a Resilience Phenotype

**Figure 3:**
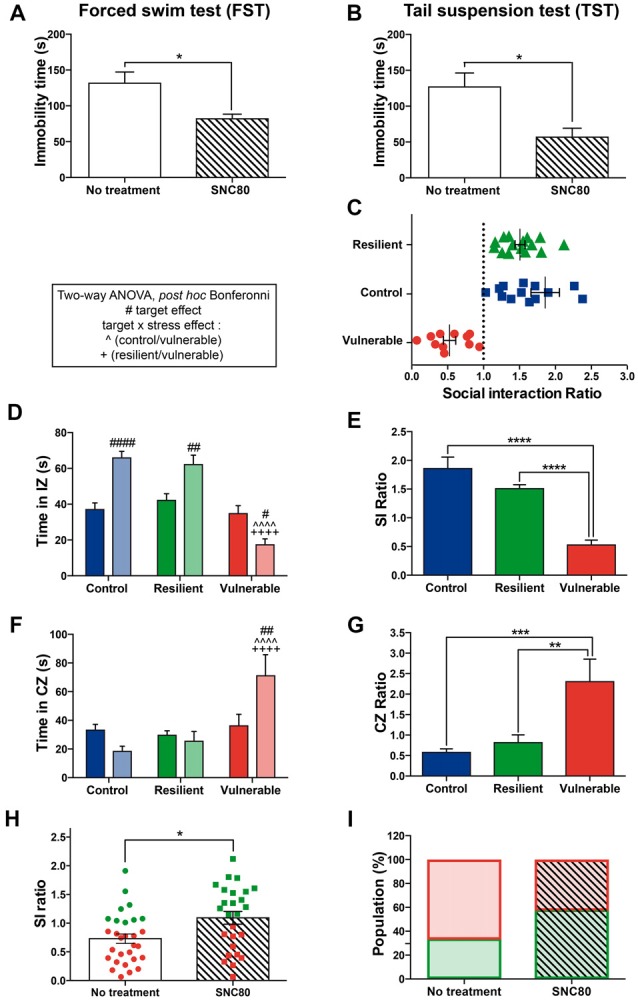
Administration of a DOPr agonist, SNC80, promotes a resilience phenotype. **(A)** Validation of SNC80 effectiveness in the forced swim test (FST). The time spent immobile is decreased with SNC80 treatment. White bar corresponds to no treatment and the streaky pattern to SNC80 treatment. Unpaired *t*-test, two tailed, *p < 0.05. *N* = 6 for each group. Error bars are mean ± SEM. **(B)** Validation of SNC80 effectiveness in the tail suspension test (TST). The time spent immobile is decreased with SNC80 treatment. White bar corresponds to no treatment and the streaky pattern to SNC80 treatment. Unpaired *t*-test, two tailed, *p < 0.05. *N* = 10 for each group. Error bars are mean ± SEM. **(C)** Distribution of SI ratio among the mouse population. N(C) = 16, N(R) = 15, N(V) = 11. Error bars are mean ± SEM. **(D)** Control and resilient mice spend more time, and vulnerable mice less time, in the IZ in the presence vs. absence of an aggressive CD1 mouse. **(E)** Vulnerable mice present a reduced SI ratio compared to control and resilient mice. **(F)** Control and resilient mice spend the same time, while vulnerable mice spend more time, in the CZs in the presence or absence of an aggressive CD1 mouse. **(G)** Vulnerable mice present an increased CZ ratio compared to control and resilient mice. For **(D,F)**: two-way ANOVA, Bonferroni *post hoc* analysis, N(C) = 16, N(R) = 15, N(V) = 11, ^#^*p* < 0.05, ^##^*p* < 0.01, ^####^, ^∧∧∧∧^,^++++^*p* < 0.0001. The two analyzed factors are the stress phenotype (blue, green and red bars for control, resilient and vulnerable mice, respectively) and the presence (pale shades) or absence (dark shades) of a CD1 mouse. ^#^for target effect, for stress × target effect, ^∧^(Control vs. Vulnerable), ^+^(Resilient vs. Vulnerable). For **(E,G)**: One-way ANOVA, Bonferroni *post hoc* analysis, N(C) = 16, N(R) = 15, N(V) = 11, **p < 0.01, ***p < 0.001, ****p < 0.0001. Error bars are mean ± SEM. **(H)** SNC80 (No treatment) during RSD increases the SI ratio compared to a classical RSD (No treatment). Green dots and squares correspond to resilient mice while the red ones represent vulnerable mice. Solid bar corresponds to no treatment and the streaky pattern to SNC80 treatment. Unpaired *t*-test, N(no treatment, NT) = 29; N(SNC80) = 26, *p < 0.05. Error bars are mean ± SEM. **(I)** Chronic treatment with SNC80, administered 1 h prior to the defeat during the 10 days of stress, increases the proportion of resilient mice after a classical protocol of RSD (No treatment). Resilient mice are represented by green squares while vulnerable ones are in red. Solid bar corresponds to no treatment and the streaky pattern to SNC80 treatment. N(NT) = 29, N(SNC80) = 26.

To study the functional role of DOPr signaling in stress resilience under the RSD in mice, the DOPr agonist SNC80 was injected 1 h prior to each social defeat session. First, we confirmed the effectiveness of 10 mg/kg SNC80 using the FST and TST: individuals treated with SNC80 showed reduced immobility time compared to saline-treated ones in both paradigms (Figure 3A: *t* = 3.635, *p* = 0.0034; Figure 3B: *t* = 3.249, *p* = 0.0045), indicating that the dose we used induced as expected antidepressant effects. The RSD paradigm was next performed on another cohort of naive mice to determine the effects of SNC80 treatment on social stress responses (Figures 3C–G). Figure 3C displays the distribution of SI ratio and Figures 3D–G the strong social avoidance behavior of vulnerable mice. Resilient and control mice displayed a SI ratio above 1 whereas vulnerable ones showed a SI ratio below 1 (Figure 3C). Two-way ANOVA was performed to address significant differences in the time spent in the IZ and CZs between stress phenotypes in the presence vs. in the absence of a CD1 mouse (Figures 3D,F). Control and resilient mice spent more time interacting with a CD1, while vulnerable ones decreased their interaction thus reducing the SI ratio (Figures 3D,E). Vulnerable mice spent more time in the CZs than control and resilient ones and thus presented a higher CZ ratio (Figures 3F,G). The SI ratio of both resilient and vulnerable mice was significantly increased when SNC80 treatment was administered throughout the RSD, compared to the SI ratio calculated without SNC80 (Figure 3H: *t* = 2.662, *p* = 0.0103). The proportion of resilient mice increased from 33% to 58% of the total population (Figure 3I), demonstrating the capacity of SNC80 to prevent the emergence of depressive-like behavior upon RSD.

### SNC80 Treatment Prevents Oxidative Stress in Hippocampal CA1 Neurons

**Figure 4:**
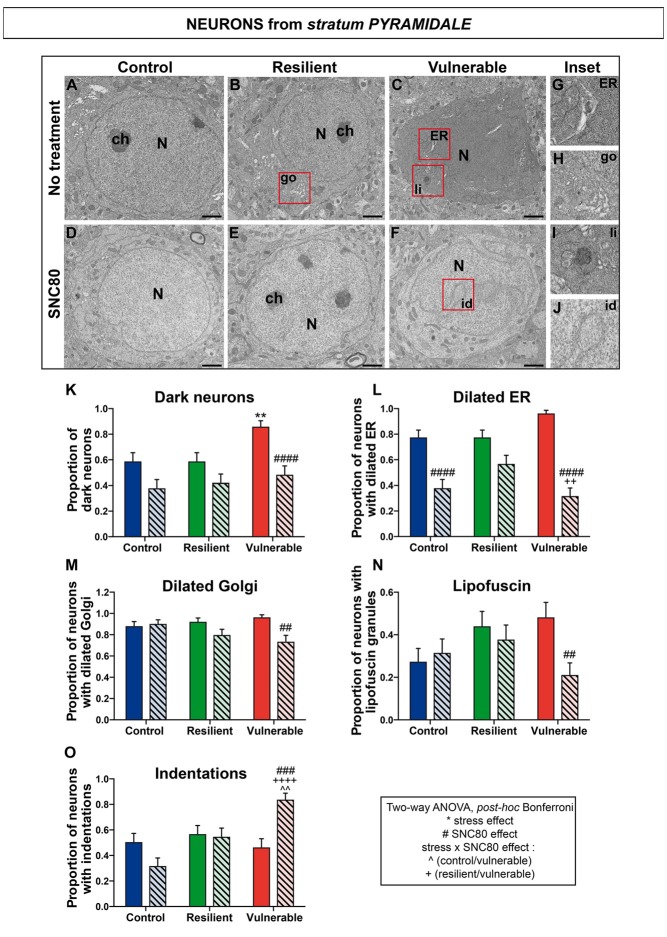
SNC80 treatment prevents oxidative stress in pyramidal cells of CA1 HPC. In **(A–C)**, representative pictures of neurons (N) in the pyramidal layer (Py) of CA1 region in ventral HPC after RSD without treatment (NT), in control, resilient and vulnerable mice, are shown respectively. In **(D–F)**, pictures of neurons from the Py layer of CA1 in vHPC after RSD with SNC80 treatment (SNC80), in control, resilient and vulnerable mice, respectively. Scale bar = 500 nm. In **(G–J)**, magnified views of dilated endoplasmic reticulum (ER), dilated Golgi apparatus (go), lipofuscin granule (li) and nuclear indentation (id), respectively. In **(K–O)**, graphs for Py layer representing the proportion of dark neurons, the proportion of neurons presenting dilated ER, dilated Golgi apparatus, lipofuscin granules and indentations, respectively, without and with SNC80 treatment. Two-way ANOVA, Bonferroni *post hoc* analysis, *N* = 48 cells for each condition. The two analyzed factors are the stress phenotype (blue, green and red bars for control, resilient and vulnerable mice under RSD, respectively) and the presence or absence of SNC80 treatment (dark blue, green and red bars for control, resilient and vulnerable mice under RSD without SNC80 treatment, respectively, and pale blue, green and red bars with a streaky pattern for control, resilient and vulnerable mice under RSD with SNC80 treatment, respectively). Significance: * for stress phenotype effect or ^#^for SNC80 treatment effect or for stress × treatment effect, ^∧^(Control vs. Vulnerable), ^+^(Resilient vs. Vulnerable) = **, ^##^, ^∧∧^*p* < 0.01, ^###^*p* < 0.001, ^####^, ^++++^*p* < 0.0001. Error bars are mean ± SEM.

**Figure 5:**
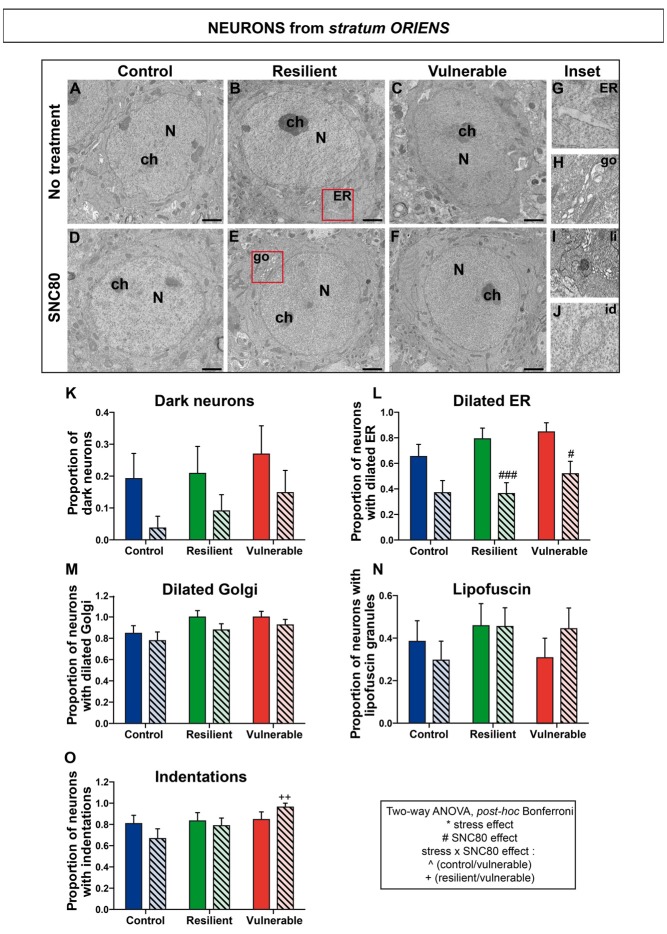
SNC80 treatment reduces oxidative stress in interneurons from *stratum oriens* of CA1 HPC. In **(A–C)**, representative pictures of neurons (N) in the *oriens* layer (Or) of CA1 region in ventral HPC after RSD without treatment (NT), in control, resilient and vulnerable mice, are shown respectively. In **(D–F)**, pictures of neurons from the Or layer of CA1 in vHPC after RSD with SNC80 treatment (SNC80), in control, resilient and vulnerable mice, respectively. Scale bar = 500 nm. In **(G–J)**, magnified views of dilated ER, dilated Golgi apparatus (go), lipofuscin granule (li), and nuclear indentation (id), respectively. In **(K–O)**, graphs for Or layer representing the proportion of dark neurons, the proportion of neurons presenting dilated ER, dilated Golgi apparatus, lipofuscin granules and indentations, respectively, without and with SNC80 treatment. Two-way ANOVA, Bonferroni *post hoc* analysis, N(NT: C; R; V) = 26; 24; 26 cells and N(SNC80: C; R; V) = 27; 33; 27 cells. The two analyzed factors are the stress phenotype (blue, green and red bars for control, resilient and vulnerable mice under RSD, respectively) and the presence or absence of SNC80 treatment (dark blue, green and red bars for control, resilient and vulnerable mice under RSD without SNC80 treatment, respectively, and pale blue, green and red bars with a streaky pattern for control, resilient and vulnerable mice under RSD with SNC80 treatment, respectively). Significance: * for stress phenotype effect or ^#^for SNC80 treatment effect or for stress × treatment effect, ^∧^(Control vs. Vulnerable), ^+^(Resilient vs. Vulnerable) = ^#^*p* < 0.05, ^++^*p* < 0.01, ^###^*p* < 0.001. Error bars are mean ± SEM.

**Figure 6:**
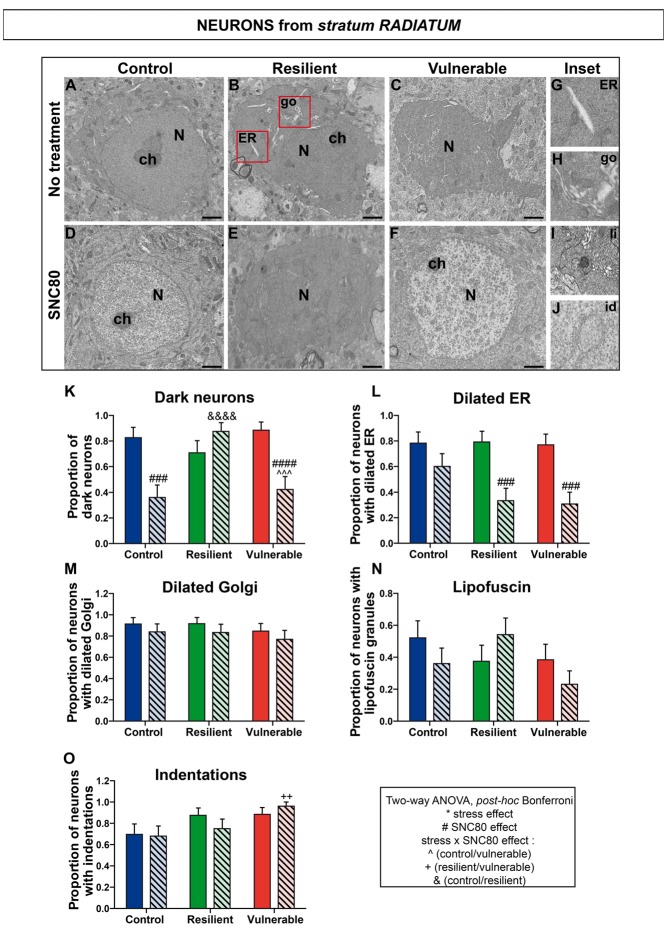
SNC80 treatment reduces oxidative stress in interneurons from *stratum radiatum* of CA1 HPC. In **(A–C)**, representative pictures of neurons (N) in the *radiatum* layer (Rad) of CA1 region in ventral HPC after RSD without treatment (NT), in control, resilient and vulnerable mice, are shown respectively. In **(D–F)**, pictures of neurons from the Rad layer of CA1 in vHPC after RSD with SNC80 treatment (SNC80), in control, resilient and vulnerable mice, respectively. Scale bar = 500 nm. In **(G–J)**, magnified views of dilated ER, dilated Golgi apparatus (go), lipofuscin granule (li), and nuclear indentation (id), respectively. In **(K–O)**, graphs for Rad layer representing the proportion of dark neurons, the proportion of neurons presenting dilated ER, dilated Golgi apparatus, lipofuscin granules and indentations, respectively, without and with SNC80 treatment. Two-way ANOVA, Bonferroni *post hoc* analysis, N(NT: C; R; V) = 23; 24; 26 cells, and N(SNC80: C; R; V) = 25; 24; 26 cells. The two analyzed factors are the stress phenotype (blue, green and red bars for control, resilient and vulnerable mice under RSD, respectively) and the presence or absence of SNC80 treatment (dark blue, green and red bars for control, resilient and vulnerable mice under RSD without SNC80 treatment, respectively, and pale blue, green and red bars with a streaky pattern for control, resilient and vulnerable mice under RSD with SNC80 treatment, respectively). Significance: * for stress phenotype effect or ^#^for SNC80 treatment effect or for stress × treatment effect, ^∧^(Control vs. Vulnerable), ^&^(Control vs. Resilient), ^+^(Resilient vs. Vulnerable) = ^++^*p* < 0.01, ^###^,^∧∧∧^*p* < 0.001, ^####^, ^&&&&^*p* < 0.0001. Error bars are mean ± SEM.

To identify mechanisms by which stimulating DOPr signaling promotes stress resilience in mice, we tested the hypothesis that SNC80 reduces oxidative stress in the ventral HPC CA1. The ventral HPC CA1 was specifically targeted considering that: (1) in the current study a significant difference of DOPr mRNA expression between resilient and vulnerable mice was only observed in this region among the entire HPC; (2) dense neuronal projections from BLA to the ventral HPC CA1 were described (Petrovich et al., 2001) as well as implicated in anxiety (Felix-Ortiz et al., 2013); and (3) this region plays a prominent role in social memory (Okuyama et al., 2016). To address this question, TEM was used to measure well-established features of cellular stress among CA1 neurons of control, resilient, and vulnerable mice, with or without SNC80 treatment. Considering that DOPr is strongly expressed by CA1 *stratum oriens* GABAergic interneurons (Stumm et al., 2004) that make synapses onto pyramidal cells and *stratum radiatum* interneurons (Maccaferri, 2005), the three neuronal subpopulations were selected for analysis. Representative images are respectively provided in Figures 4A–J (*stratum pyramidale*), Figures 5A–J (*stratum oriens*) and Figures 6A–J (*stratum radiatum*).

Two-way ANOVA was systematically performed to address significant differences in oxidative features between stress phenotypes in the presence vs. in the absence of SNC80 treatment (Figures 4, 5, 6). The condensation of cytoplasmic and nucleoplasmic contents, resulting in a “dark” appearance in EM, has been associated with the shrinkage of cells undergoing oxidative stress (Oster-Granite et al., 1996; Tremblay et al., 2012; Bisht et al., 2016b). ANOVA analysis of dark neurons revealed main effects of stress phenotype (Figure 4K: *F*_(2,282)_ = 4.453, *p* = 0.0125) and SNC80 treatment on their prevalence in the CA1 *stratum pyramidale* (Figure 4K: *F*_(1,282)_ = 19.76, *p* < 0.0001). *Post hoc* analysis especially revealed an increased prevalence of dark pyramidal cells in vulnerable mice compared to resilient and non-stressed ones (Figure 4K). After treatment with SNC80, this increased prevalence observed upon stress in vulnerable mice was prevented (Figure 4K). ANOVA further identified treatment effects in both *strata oriens* (Figure 5K: *F*_(1,157)_ = 5.403, *p* = 0.0214) and *radiatum* (Figure 6K: *F*_(1,142)_ = 13.15, *p* = 0.0004), and a stress phenotype by treatment interaction in *stratum radiatum* (Figure 6K: *F*_(2,142)_ = 8.918, *p* = 0.0002). In particular, control and vulnerable mice treated with SNC80 displayed a decreased prevalence of dark interneurons in *stratum radiatum* compared to non-treated control and vulnerable mice (Figure 6K). However, *post hoc* analysis did not reveal significant differences in *stratum oriens* (Figure 5K). Surprisingly, control mice, which were housed in the room where RSD occurred, displayed a significant proportion of “dark neurons” in *stratum radiatum* (Figure 6K) suggesting some degree of stress in these animals witnessing the social defeat.

The analysis of neurons with a dilated ER, considered the best-characterized feature of cellular stress at the ultrastructural level (Schönthal, 2012), revealed main effects for both treatment (Figure 4L: *F*_(1,282)_ = 67.46, *p* < 0.0001) and interaction (Figure 4L: *F*_(2,282)_ = 6.240, *p* < 0.0022) in *stratum pyramidale*, and treatment effects in *strata oriens* (Figure 5L: *F*_(1,157)_ = 22.43, *p* < 0.0001) and *radiatum* (Figure 6L: *F*_(1,142)_ = 24.12, *p* < 0.0001). Precisely, the proportion of neurons with a dilated ER was reduced by SNC80 treatment in *stratum pyramidale* from control and vulnerable mice compared to non-treated control and vulnerable ones (Figure 4L). For the interneurons, this reduction induced by SNC80 was observed in resilient and vulnerable mice (*stratum oriens*, Figure 5L and *stratum radiatum*, Figure 6L).

The proportion of neurons displaying a dilated Golgi apparatus, documented as another cellular stress marker (Welch and Suhan, 1985), revealed main treatment (Figure 4M: *F*_(1,282)_ = 7.664, *p* = 0.0060) and interaction (Figure 4M: *F*_(2,282)_ = 3.263, *p* = 0.0397) effects in pyramidal cells. A main stress phenotype effect was also observed in *stratum oriens* (Figure 5M: *F*_(2,157)_ = 4.109, *p* < 0.0001), but failed to reach significance in *stratum radiatum* (Figure 6M). *Post hoc* analysis revealed a decreased proportion of neurons displaying a dilated Golgi apparatus upon SNC80 treatment in *stratum pyramidale* of vulnerable mice (Figure 4M). No significant differences were observed for interneurons of *oriens* and *radiatum* layers (Figures 5M, 6M).

Finally, quantitative analyses of lipofuscin granules, i.e., cytoplasmic granules resulting from the oxidation of unsaturated fatty acids (Sohal and Wolfe, 1986) which are related to cellular aging (Sohal and Wolfe, 1986), were conducted. Although stress phenotype and treatment effects failed to reach statistical significance in ANOVA for all layers (Figures 4N, 5N, 6N), the number of neurons showing lipofuscin granules was found to be significantly reduced upon *post hoc* analyses in pyramidal cells of SNC80-treated vulnerable mice compared to non-treated ones (Figure 4N).

Nuclear indentations, also related to cellular aging (Roos and Bots, 1983), displayed a main stress phenotype effect (Figure 4O: *F*_(2,282)_ = 6.106, *p* = 0.0025) and a main stress phenotype by treatment effect in *stratum pyramidale* (Figure 4O: *F*_(2,282)_ = 8.742, *p* = 0.0002) while only a stress phenotype effect was identified in *stratum radiatum* (Figure 6O: *F*_(2,142)_ = 4.656, *p* = 0.0110). Thus, treated vulnerable mice exhibited an increased prevalence of neurons presenting indentations compared to treated resilient and control mice in *stratum pyramidale* (Figure 4O). Moreover, the prevalence of neurons showing nuclear indentations was specifically increased in *stratum pyramidale* of treated vulnerable mice vs. non-treated ones (Figure 4O). Finally, *post hoc* analyses revealed a significant increase of *strata oriens* and *radiatum* interneurons with indentations in treated vulnerable mice compared to treated controls (Figures 5O, 6O).

Tables are provided in Supplemental information presenting values for the two-way ANOVAs followed by Bonferroni *post hoc* analyses for each analyzed layer (Supplementary Tables S2, S3, S4). Overall, these findings suggest that SNC80 treatment prevents the occurrence of cellular stress among CA1 pyramidal cells and interneurons, ultimately promoting resilience to chronic social stress.

## Discussion

Elucidating the underlying mechanisms of resilience is a pressing medical challenge in the 21st century, especially considering the devastating outcomes of chronic stress on major depression (Davidson and Mcewen, 2012), cognitive aging, and neurodegenerative diseases (Green et al., 2003; Modrego, 2010). The aim of this study was to investigate the involvement of opioid ENK signaling in the development of stress resilience in mice.

The RSD paradigm was selected to reproduce the unpredictable disruptions of daily life and study the underlying mechanisms of stress resilience. As expected from previous RSD studies in mice (Golden et al., 2011), we obtained 33% of animals displaying a resilient phenotype among the total population. To validate our paradigm, we showed that vulnerable and resilient mice demonstrated an increase of their plasmatic corticosterone levels. RSD is considered the most demanding paradigm in terms of hypothalamic-pituitary-adrenal axis activation (Koolhaas et al., 1997). When comparing control and defeated rodents, an important variation of plasmatic corticosterone levels was previously reported throughout RSD, with a peak measured during the first and the last defeat days in mice and with a return to basal levels appearing 1 week after (Keeney et al., 2006). Krishnan et al. (2007) obtained a result comparable to ours where vulnerable mice as well as resilient ones had an increase in plasma corticosterone levels on the day following the last defeat.

Our results indicate that mRNA levels of ENK decrease in the BLA of vulnerable mice compared to resilient ones without significant differences between resilient and control animals. Previous studies demonstrated a similar reduction of ENKs mRNA levels in the BLA of vulnerable rats, while inactivation of ENKs in the same area reproduced a vulnerability phenotype (Bérubé et al., 2013, 2014). These findings provide support to the hypothesis that BLA-ENK neurotransmission contributes to the development of stress resilience in both rats and mice, raising the intriguing possibility that a similar role might be exerted in humans.

Reduced mRNA levels of ENK (in the BLA) under the RSD paradigm were also associated to a decreased mRNA expression of its DOPr (in BLA-targeted CA1 *stratum oriens* of ventral HPC) in vulnerable mice only. These findings suggest that signaling between BLA-ENK projections and their targets, DOPr-expressing neurons in the CA1 *stratum oriens* of ventral HPC, is preserved during resilience to social stress. Considering that changes in mRNA signals do not necessarily reflect the protein levels (Hatzimanikatis et al., 1999), additional experiments determining the changes in ENK and DOPr protein expression are however warranted to provide a comprehensive picture of their association with stress resilience. In addition, several other, possibly indirect, neuroanatomical connections of non-enkephalinergic neurochemical nature such as glutamatergic projections (Felix-Ortiz et al., 2013; Rei et al., 2015) may have contributed to this phenomenon. Also considering that social stress recruits several brain regions including classical ones mediating the stress response, comprising the hypothalamus, septum, bed nucleus of stria terminalis, preoptic area and some nuclei in brainstem, as shown by mapping experiments with the immediate-early gene c-fos (Martinez et al., 2002), and that DOPr is widely expressed across the brain (Scherrer et al., 2006), it would be important in future experiments to investigate the role of additional brain areas.

Pharmacological activation of DOPr with the agonist SNC80 throughout RSD further increased the proportion of resilient animals, indicating that the endogenous ENK-DOPr system could be targeted to promote coping behavior and adaptation in the face of chronic social adversity. ENKs are known to reduce stress-induced neuroendocrine and autonomic responses, and also to stimulate these effector systems under normal, non-stressful conditions. A distinctive feature of ENKs analgesic action is their blunting of pain’s distressing, affective component, without dulling the sensation itself. Therefore, our findings propose that ENK-DOPr signaling may diminish the impact of social stress on depressive-like behavior by attenuating physiological responses during resilience, including emotional and affective states, leading to reduced anxiety. Indeed, the results from several rodent studies consistently indicate that ENK-DOPr signaling mainly exerts anxiolytic effects (Drolet et al., 2001; Gendron et al., 2015; Henry et al., 2017).

To provide insights into the underlying mechanisms, considering that anxiety levels in mice are tightly related to antioxidant enzymes expression (Hovatta et al., 2005), ultrastructural analyses of oxidative damage to neurons were conducted in the CA1 region (*strata oriens*, *pyramidale*, *radiatum*) of ventral HPC. Electron density was first examined considering that the condensation of cytoplasmic and nucleoplasmic contents is strongly associated with oxidative challenges to neurons (Peters et al., 1998) and glial cells (Bisht et al., 2016b,c). Dark neurons were previously described in elderly monkeys (Peters et al., 1998) as well as rodent models of Huntington’s and Alzheimer’s diseases (Oster-Granite et al., 1996; Turmaine et al., 2000; Yang et al., 2008), ischemia (Kirino et al., 1984), epilepsy (Atillo et al., 1983) and aging (Tremblay et al., 2012). In our analyses, social stress and SNC80 were found to exert different effects on the electron density between neuronal subpopulations. Among these, dark pyramidal cells became more prevalent in vulnerable mice compared to control and resilient ones, while SNC80 treatment prevented this increase. Stimulating ENK-DOPr signaling may thus reduce the deleterious impact of chronic social stress by preventing oxidative damage.

This finding is supported by the reduced prevalence of dilated ER that we measured upon SNC80 treatment in CA1 interneurons and pyramidal cells from the experimental groups analyzed. The best-characterized sign of cellular disturbance at the ultrastructural level is ER stress, since the pathological accumulation of unfolded or misfolded proteins makes its lumen appear dilated in EM (Malhotra and Kaufman, 2007; Ron and Walter, 2007; Schönthal, 2012). The unfolded protein response (UPR) is triggered upon ER stress to stop protein translation, degrade unfolded proteins or activate signaling pathways leading to refolding (Ron and Walter, 2007). During the refolding process, reactive oxygen species are produced, leading to impaired redox balance resulting in oxidative stress. Since the refolding process depends on redox homeostasis, oxidative stress can disrupt proper folding mechanisms thus exacerbating further the ER stress. Similarly, dilation of Golgi cisternae, also associated with oxidative stress (Welch and Suhan, 1985), was found to be prevented in pyramidal cells of SNC80-treated vulnerable mice. Whether SNC80 treatment decreases ER and Golgi stress directly, or preserves homeostasis by activating the UPR pathway, among others, remains to be investigated.

Additional signs of cellular stress were also reduced upon stimulation of DOPr signaling, such as the prevalence of lipofuscin granules, which are long-considered the major hallmark of cellular aging (Sohal and Wolfe, 1986), in pyramidal cells of SNC80-treated vulnerable mice. This finding indicates that pharmacological treatment with DOPr agonists might prevent the accelerated-aging effect induced by stress (Green et al., 2003). Additionally, nuclear indentations became more frequent in pyramidal cells and interneurons of vulnerable mice treated with SNC80. Considering that nuclear indentations have been associated with the remodeling of cellular morphology (Versaevel et al., 2014), their appearance might reflect an increased neuronal plasticity induced by SNC80 treatment. Indeed, DOPr are known to induce long-term plasticity of hippocampal GABAergic synapses (Rozov et al., 2017).

Overall, this study suggests that ENK/DOPr signaling between the BLA and CA1 (*stratum oriens*) of ventral HPC is preserved in resilient mice and impaired in vulnerable ones under the RSD paradigm. Moreover, this study highlighted a key role of DOPr signaling in promoting social stress resilience through its protective effect against cellular stress among excitatory and inhibitory neurons of the ventral HPC CA1. Additional research focusing on the functional role of DOPr in preventing cellular stress in the face of adversity may provide novel insights into how the intimate relationship between oxidative stress and psychosocial stress influences resilience. The current work underlines the importance of studying endogenous opioids as novel therapeutic targets for stress-related disorders, and suggests that pharmacological activation of DOPr could be an interesting candidate to prevent the exacerbation of cellular stress induced by psychosocial stress.

## Author Contributions

GD, MET, MSH, NV and LG designed the study. MSH, KB and LG conducted experiments and analyzed data. MSH prepared the figures. AT-B provided expertise. All the authors contributed to writing the manuscript.

## Conflict of Interest Statement

The authors declare that the research was conducted in the absence of any commercial or financial relationships that could be construed as a potential conflict of interest.
