# Supplementary material for: Delta Opioid Receptor Signaling Promotes Resilience to Stress Under the Repeated Social Defeat Paradigm in Mice
Source: Front Mol Neurosci. 2018 Apr 6;11:100. doi: 10.3389/fnmol.2018.00100 (PMC5897549; doi:10.3389/fnmol.2018.00100)
Supplement: Supplementary file 1 [file Table_1.pdf]

Bregma Levels in Hippocampus	Regions	Layers	Control	Resilient	Vulnerable	p values
Dorsal Hippocampus (Bregma = -2.06 mm)	CA1	*Pyramidal*	0.608	0.590	0.576	0.8179
		Radiatum	0.232	0.249	0.265	0.6941
		Oriens	0.323	0.283	0.326	0.642
	CA3	*Pyramidal*	0.513	0.467	0.498	0.7778
		Radiatum	0.215	0.230	0.230	0.42
		Oriens	0.270	0.259	0.248	0.9277
Central Hippocampus (Bregma = -2.70 mm)	CA1	*Pyramidal*	0.491	0.492	0.495	0.8551
		Radiatum	0.196	0.217	0.207	0.752
		Oriens	0.260	0.256	0.274	0.8788
	CA3	*Pyramidal*	0.561	0.542	0.568	0.8637
		Radiatum	0.224	0.233	0.249	0.6991
		Oriens	0.235	0.247	0.276	0.157
Ventral Hippocampus (Bregma = -2.92 mm)	CA1	*Pyramidal*	0.464	0.484	0.458	0.7607
		Radiatum	0.194	0.219	0.223	0.5551
		Oriens	**0.271**	**0.275**	**0.169**	**0.0022**
	CA3	*Pyramidal*	0.502	0.534	0.515	0.7921
		Radiatum	0.224	0.248	0.240	0.6897
		Oriens	0.219	0.248	0.249	0.3355
	SUBv		0.368	0.467	0.406	0.1962
