# Supplementary material for: Delta Opioid Receptor Signaling Promotes Resilience to Stress Under the Repeated Social Defeat Paradigm in Mice
Source: Front Mol Neurosci. 2018 Apr 6;11:100. doi: 10.3389/fnmol.2018.00100 (PMC5897549; doi:10.3389/fnmol.2018.00100)
Supplement: Supplementary file 2 [file Table_2.PDF]

MARKERS		F(DFn, DFd)	p values
Dark cells	**INTERACTION**	F (2, 282) = 1,281	P = 0,2794
	**STRESS**	F (2, 282) = 4,453	**P = 0,0125**
	**TREATMENT**	F (1, 282) = 19,76	**P < 0,0001**
Dilated ER	**INTERACTION**	F (2, 282) = 6,240	**P = 0,0022**
	**STRESS**	F (2, 282) = 1,181	P = 0,3086
	**TREATMENT**	F (1, 282) = 67,46	**P < 0,0001**
Dilated Golgi	**INTERACTION**	F (2, 282) = 3,263	**P = 0,0397**
	**STRESS**	F (2, 282) = 0,3892	P = 0,6780
	**TREATMENT**	F (1, 282) = 7,664	**P = 0,0060**
Lipofuscin	**INTERACTION**	F (2, 282) = 2,731	P = 0,0669
	**STRESS**	F (2, 282) = 1,420	P = 0,2434
	**TREATMENT**	F (1, 282) = 3,058	P = 0,0814
Indentations	**INTERACTION**	F (2, 282) = 8,742	**P = 0,0002**
	**STRESS**	F (2, 282) = 6,106	**P = 0,0025**
	**TREATMENT**	F (1, 282) = 0,9697	P = 0,3256

	**No treatment**			**SNC80**			**Interaction stress x SNC80**		
	Control vs Resilient	Control vs Vulnerable	Resilient vs Vulnerable	Control	Resilient	Vulnerable	Control vs Resilient	Control vs Vulnerable	Resilient vs Vulnerable
**Significance?**	No	**Yes**	**Yes**	No	No	**Yes**	No	No	No
**t values**	0.000	**2.781**	**2.781**	2.139	1.710	**3.850**	0.428	1.070	0.642
**p values**	1	**0.0058**	**0.0058**	0.0333	0.0884	**0.0001**	0.6690	0.2855	0.5214
**Significance?**	No	No	No	**Yes**	No	**Yes**	No	No	**Yes**
**t values**	0.000	2.134	2.134	**4.506**	2.371	**7.352**	2.134	0.711	**2.845**
**p values**	1	0.0337	0.0337	**0.0001**	0.0184	**0.0001**	0.0337	0.4777	**0.0048**
**Significance?**	No	No	No	No	No	**Yes**	No	No	No
**t values**	0.600	1.198	0.598	0.299	1.798	**3.295**	1.497	2.396	0.899
**p values**	0.549	0.2319	0.5503	0.7652	0.0732	**0.0011**	0.1355	0.0172	0.3694
**Significance?**	No	No	No	No	No	**Yes**	No	No	No
**t values**	1.731	2.164	0.433	0.433	0.649	**2.814**	0.649	1.082	1.731
**p values**	0.0845	0.0313	0.6653	0.6653	0.5169	**0.0052**	0.5169	0.2802	0.0845
**Significance?**	No	No	No	No	No	**Yes**	No	**Yes**	**Yes**
**t values**	0.640	0.427	1.066	1.919	0.213	**3.837**	2.345	**5.329**	**2.984**
**p values**	0.5227	0.6697	0.2873	0.056	0.8315	**0.0002**	0.0197	**0.0001**	**0.0031**
