# Supplementary material for: Delta Opioid Receptor Signaling Promotes Resilience to Stress Under the Repeated Social Defeat Paradigm in Mice
Source: Front Mol Neurosci. 2018 Apr 6;11:100. doi: 10.3389/fnmol.2018.00100 (PMC5897549; doi:10.3389/fnmol.2018.00100)
Supplement: Supplementary file 3 [file Table_3.PDF]

| MARKERS | | F(DFn, DFd) | p values |
|---|---|---|---|
| | INTERACTION | F (2, 157) = 0,04531 | P = 0,9557 |
| | STRESS | F (2, 157) = 0,9301 | P = 0,3967 |
| Dark cells | TREATMENT | F (1, 157) = 5,403 | **P = 0,0214** |
| | INTERACTION | F (2, 157) = 0,3465 | P = 0,7077 |
| | STRESS | F (2, 157) = 1,810 | P = 0,1671 |
| Dilated ER | TREATMENT | F (1, 157) = 22,43 | **P < 0,0001** |
| | INTERACTION | F (2, 157) = 0,1345 | P = 0,8743 |
| | STRESS | F (2, 157) = 4,109 | **P = 0,0182** |
| Dilated Golgi | TREATMENT | F (1, 157) = 3,648 | P = 0,0580 |
| | INTERACTION | F (2, 157) = 0,7054 | P = 0,4955 |
| | STRESS | F (2, 157) = 0,7926 | P = 0,4545 |
| Lipofuscin | TREATMENT | F (1, 157) = 0,03681 | P = 0,8481 |
| | INTERACTION | F (2, 157) = 1,522 | P = 0,2215 |
| | STRESS | F (2, 157) = 2,520 | P = 0,0837 |
| Indentations | TREATMENT | F (1, 157) = 0,1471 | P = 0,7018 |

| | No treatment | | | SNC80 | | | Interaction stress x SNC80 | | |
|---|---|---|---|---|---|---|---|---|---|
| | Control vs Resilient | Control vs Vulnerable | Resilient vs Vulnerable | Control | Resilient | Vulnerable | Control vs Resilient | Control vs Vulnerable | Resilient vs Vulnerable |
| Significance? | No | No | No | No | No | No | No | No | No |
| t values | 0.158 | 0.773 | 0.600 | 1.575 | 1.220 | 1.229 | 0.579 | 1.137 | 0.614 |
| p values | 0.8747 | 0.4407 | 0.5494 | 0.1173 | 0.2243 | 0.2209 | 0.5634 | 0.2573 | 0.5401 |
| Significance? | No | No | No | No | **Yes** | No | No | No | No |
| t values | 1.048 | 1.493 | 0.414 | 2.220 | **3.434** | 2.567 | 0.056 | 1.171 | 1.285 |
| p values | 0.2962 | 0.1374 | 0.6794 | 0.0279 | **0.0008** | 0.0112 | 0.9554 | 0.2434 | 0.2007 |
| Significance? | No | No | No | No | No | No | No | No | No |
| t values | 1.858 | 1.897 | 0.000 | 0.851 | 1.545 | 0.922 | 1.331 | 1.861 | 0.621 |
| p values | 0.065 | 0.0597 | 1 | 0.3961 | 0.1244 | 0.3579 | 0.1851 | 0.0646 | 0.5355 |
| Significance? | No | No | No | No | No | No | No | No | No |
| t values | 0.528 | 0.563 | 1.079 | 0.652 | 0.029 | 1.009 | 1.237 | 1.104 | 0.079 |
| p values | 0.5982 | 0.5742 | 0.2822 | 0.5154 | 0.9769 | 0.3145 | 0.2179 | 0.2713 | 0.9371 |
| Significance? | No | No | No | No | No | No | No | **Yes** | No |
| t values | 0.235 | 0.361 | 0.118 | 1.334 | 0.440 | 1.105 | 1.214 | **2.830** | 1.754 |
| p values | 0.8145 | 0.7186 | 0.9062 | 0.1841 | 0.6605 | 0.2709 | 0.2266 | **0.0053** | 0.0814 |
