# Supplementary material for: Delta Opioid Receptor Signaling Promotes Resilience to Stress Under the Repeated Social Defeat Paradigm in Mice
Source: Front Mol Neurosci. 2018 Apr 6;11:100. doi: 10.3389/fnmol.2018.00100 (PMC5897549; doi:10.3389/fnmol.2018.00100)
Supplement: Supplementary file 4 [file Table_4.PDF]

MARKERS		F(DFn, DFd)	p values
Dark cells	**INTERACTION**	F (2, 142) = 8,918	**P = 0,0002**
	**STRESS**	F (2, 142) = 2,758	P = 0,0668
	**TREATMENT**	F (1, 142) = 13,15	**P = 0,0004**
Dilated ER	**INTERACTION**	F (2, 142) = 1,507	P = 0,2251
	**STRESS**	F (2, 142) = 1,599	P = 0,2057
	**TREATMENT**	F (1, 142) = 24,12	**P < 0,0001**
Dilated Golgi	**INTERACTION**	F (2, 142) = 0,002508	P = 0,9975
	**STRESS**	F (2, 142) = 0,6046	P = 0,5477
	**TREATMENT**	F (1, 142) = 1,728	P = 0,1908
Lipofuscin	**INTERACTION**	F (2, 142) = 1,789	P = 0,1709
	**STRESS**	F (2, 142) = 1,440	P = 0,2404
	**TREATMENT**	F (1, 142) = 0,3817	P = 0,5377
Indentations	**INTERACTION**	F (2, 142) = 0,8587	P = 0,4259
	**STRESS**	F (2, 142) = 4,656	**P = 0,0110**
	**TREATMENT**	F (1, 142) = 0,1124	P = 0,7379

	**No treatment**			**SNC80**			**Interaction stress x SNC80**		
	Control vs Resilient	Control vs Vulnerable	Resilient vs Vulnerable	Control	Resilient	Vulnerable	Control vs Resilient	Control vs Vulnerable	Resilient vs Vulnerable
**Significance?**	No	No	No	**Yes**	No	**Yes**	Yes	No	**Yes**
**t values**	0.950	0.481	1.466	**3.796**	1.359	**3.915**	4.241	0.530	**3.757**
**p values**	0.3437	0.6313	0.1449	**0.0002**	0.1763	**0.0001**	0.0001	0.5969	**0.0003**
**Significance?**	No	No	No	No	**Yes**	**Yes**	No	No	No
**t values**	0.069	0.103	0.175	1.390	**3.492**	**3.659**	2.052	2.295	0.199
**p values**	0.9451	0.9181	0.8613	0.1667	**0.0006**	**0.0004**	0.0420	0.0232	0.8425
**Significance?**	No	No	No	No	No	No	No	No	No
**t values**	0.035	0.649	0.693	0.703	0.804	0.772	0.065	0.703	0.630
**p values**	0.9721	0.5174	0.4894	0.4832	0.4227	0.4414	0.9483	0.4832	0.5297
**Significance?**	No	No	No	No	No	No	No	No	No
**t values**	1.030	0.981	0.069	1.146	1.183	1.136	1.302	0.945	2.249
**p values**	0.3048	0.3283	0.9451	0.2537	0.2388	0.2579	0.1950	0.3463	0.0261
**Significance?**	No	No	No	No	No	No	No	**Yes**	No
**t values**	1.596	1.714	0.088	0.141	1.125	0.720	0.636	**2.611**	1.941
**p values**	0.1127	0.0887	0.9300	0.8881	0.2625	0.4727	0.5258	**0.0100**	0.0542
