# Supplementary material for: Delta Opioid Receptor Signaling Promotes Resilience to Stress Under the Repeated Social Defeat Paradigm in Mice
Source: Front Mol Neurosci. 2018 Apr 6;11:100. doi: 10.3389/fnmol.2018.00100 (PMC5897549; doi:10.3389/fnmol.2018.00100)
Supplement: Supplementary file 5 [file Data_Sheet_1.docx]

**Supplementary Information**

**Figure S1. Summary of experiments.**

Schematic representation of animal cohorts including the number of animals used for each experiment.

**Figure S2. Sense probe vs. antisense probe DOPr.**

Magnified views of brain sections (Bregma -1.34mm and -2.80mm) tested with sense probe *versus* antisense probe for DOPr. Scale bar = 1mm.

**Table S1. Quantification of DOPr mRNA levels across hippocampus after repeated social defeat stress.**

Mean values for all regions in hippocampus quantified for DOPr mRNA levels. Dorsal (Bregma 2.06mm), central (Bregma -2.70mm) and ventral hippocampus (Bregma -3.08mm) were quantified in CA1 and CA3 regions in three different *strata* (*pyramidale*, *oriens* and *radiatum*) as well as in ventral subiculum (SUBv). Bold numbers correspond to the only data showing significant differences between groups.

**Table S2. Statistical analysis (two-way ANOVA with Bonferroni *post-hoc* analysis) for prevalence of different oxidative stress markers in *stratum pyramidale* described by electron microscopy.** Left part of the table provides statistical data of main effects and the right part (purple) provides statistical data for Bonferroni *post-hoc*. Bold numbers are considered as significant.

**Table S3. Statistical analysis (two-way ANOVA with Bonferroni *post-hoc* analysis) for prevalence of different oxidative stress markers in *stratum oriens* described by electron microscopy.** Left part of the table provides statistical data of main effects and the right part (purple) provides statistical data for Bonferroni *post-hoc*. Bold numbers are considered as significant.

**Table S4. Statistical analysis (two-way ANOVA with Bonferroni *post-hoc* analysis) for prevalence of different oxidative stress markers in *stratum radiatum* described by electron microscopy.** Left part of the table provides statistical data of main effects and the right part (purple) provides statistical data for Bonferroni *post-hoc*. Bold numbers are considered as significant.
